# Conductive Poly(vinyl alcohol)/Multiwalled Carbon Nanotubes Nanofiber Membranes with High Environmental Stability

**DOI:** 10.1021/acsomega.5c12610

**Published:** 2026-02-05

**Authors:** Hui Xiao, Hongyu Lin, Jingyi Wang, Chuanli Yu, Huaxin Wang, Liqun Chen, Hongbing Jia

**Affiliations:** 1 School of New Materials and Shoes & Clothing Engineering, 384337 Liming Vocational University, Quanzhou 362000, China; 2 Key Laboratory for Soft Chemistry and Functional Materials of Ministry of Education, 12436 Nanjing University of Science and Technology, Nanjing 210094, China; 3 HTT Material Technology CO. Ltd., Quanzhou 362005, China

## Abstract

Poly­(vinyl alcohol) (PVA) has good processability and design flexibility, making its conductive nanofiber membranes promising for bioelectronics and regenerative medicine. However, the hydrophilic nature of PVA results in poor water stability, which disrupts the conductive network and limits the environmental adaptability. In this study, conductive PVA nanofiber membranes with uniformly dispersed multiwalled carbon nanotubes (MWCNTs) were fabricated via electrospinning. The micromorphology and electrical and mechanical properties of nanofiber membranes were investigated. The results show that conductive pathways are established in nanofiber membranes by incorporating MWCNTs. Compared with pure PVA nanofiber membranes, those containing MWCNTs exhibit lower surface resistivity and improved mechanical properties. At a loading of 1.6 wt % MWCNTs, the membrane displayed a reduction in surface resistivity by 4 orders of magnitude (6.65 × 10^7^ Ω) and an increase in tensile strength by a factor of 3.2 (8.26 MPa). The resulting nanofiber membranes demonstrate excellent stability and durability upon exposure to acidic and alkaline media, thermal variations, and ultraviolet radiation. This study provides a viable strategy for the large-scale fabrication of nanofiber membranes for use in artificial nerve conduits.

## Graphical abstract

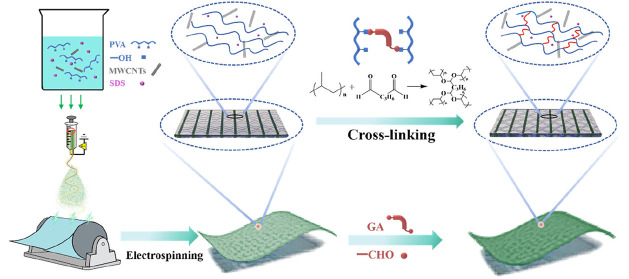

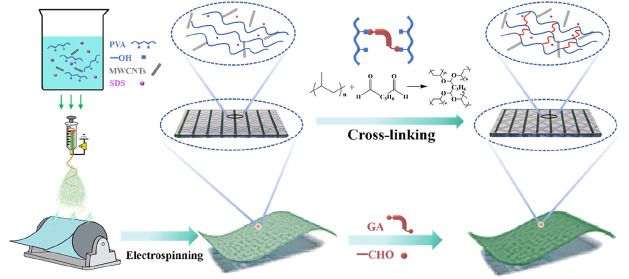

## 1 Introduction

With the increasing number of patients suffering from nerve injuries each year, artificial nerve conduits are being utilized to bridge the gap between severed nerve ends, aiming to promote nerve regeneration and subsequent functional recovery.^1^ Current research focuses on the development of artificial nerve conduits that meet essential criteria, including biocompatibility, safety, the provision of a stable conductive microenvironment, and suitable mechanical strength, etc.^2–4^

Polymeric nanofiber membranes have attracted extensive attention owing to their high specific surface area, controllable structures, and outstanding physicochemical properties.^5–8^ Among the polymers used, poly­(vinyl alcohol) (PVA), a water-soluble synthetic polymer, is frequently employed for nanofiber fabrication because of its excellent film-forming ability, biocompatibility, biodegradability, and relatively high mechanical strength.^9,10^ Nevertheless, pristine PVA exhibits intrinsic limitations, including low electrical conductivity, poor thermal stability, and limited resistance to acidic and alkaline conditions, which hinder its application in functional materials requiring superior electrical performance and environmental robustness.^11–14^ Consequently, the development of conductive PVA nanofiber membranes with enhanced environmental stability has become a major research focus.^15–17^

Multiwalled carbon nanotubes (MWCNTs) are widely investigated as polymer reinforcements due to their high aspect ratio, superior mechanical strength, and remarkable structural stability.^18–20^ These features enable the formation of efficient conductive networks at low loadings, thereby enhancing both electrical conductivity and mechanical performance while ensuring long-term stability under harsh operating conditions. Despite their great potential, practical utilization of MWCNTs is often constrained by challenges in achieving a homogeneous dispersion and controlled orientation. Surface modification and functionalization are typically adopted to address these issues. For example, Moon et al.^21^ reported that polydopamine-coated MWCNTs significantly improved the mechanical, thermal, optical, and antibacterial properties of PVA nanocomposite films compared with pristine or acid-treated CNTs/PVA systems. Similarly, Farag and Abdel-Fattah^22^ demonstrated that plasma-functionalized MWCNTs (f-MWCNTs) had stronger interfacial compatibility with PVA. However, these strategies usually involve complex processing steps or additional chemicals, which complicate fabrication and may impair the intrinsic properties of MWCNTs. Furthermore, repeated washing or exposure to acidic/alkaline environments may lead to aggregation and migration of functionalized fillers, ultimately reducing the conductivity and environmental durability. Therefore, achieving conductive nanofiber membranes with both high environmental stability and facile processability remains a challenge.

Electrospinning offers a straightforward and versatile approach to nanofiber fabrication, relying on high-voltage electrostatic fields to transform polymer solutions into continuous fibers.^23–25^ In this work, conductive PVA/MWCNTs nanofiber membranes were successfully fabricated via a simple one-step solution electrospinning process, followed by cross-linking with glutaraldehyde (GA). The morphology, internal architecture, and mechanical and electrical properties of the resulting nanofiber membranes were systematically investigated. By combining MWCNTs-based mechanical reinforcement and conductive pathway formation with GA-induced cross-linking network, this study establishes a rational design strategy for the fabrication of conductive nanofiber membranes with balanced mechanical strength and structural stability.

## 2 Materials and Methods

### 2.1 Materials

PVA­(M_w_ = 195000) and sodium lauryl sulfate (SDS) were obtained from Shanghai Macklin Biochemical Technology Co., China. Hydrochloric acid (HCl, 38 wt %) was supplied by Nanjing Chemical Reagent Co., China. Glutaraldehyde (GA, 50 wt %) was supplied by Shanghai Aladdin Biochemical Technology Co., China. Acetone was supplied by Shanghai Lingfeng Chemical Reagent Co., China. MWCNTs (length 0.5–2 μm) was obtained from Nanjing Xianfeng Nanomaterial Technology Co., China.

### 2.2 Preparation of Nanofiber Membranes

MWCNTs (0.32, 0.48, and 0.64 g) was dispersed in deionized water containing 0.01 g of SDS as a dispersant, followed by ultrasonication for 3 h to obtain a stable and homogeneous dispersion. PVA was then added, and the mixture was stirred in an oil bath at 95 °C for 3 h. After degassing to remove bubbles, electrospinning solutions were prepared with a fixed 7 wt % PVA concentration and MWCNTs loadings of 0.8, 1.2, and 1.6 wt %.

The solution was loaded into a microsyringe pump and electrospun for 10 h under the following conditions: an ambient temperature of 25 °C, a relative humidity of 55%, an applied voltage of 14 kV, a feed rate of 0.5 mL/h, and a tip-to-collector distance of 14 cm.

The obtained nanofiber membranes were immersed in a 0.5 M GA solution in acetone, and HCl was added to catalyze the cross-linking reaction for 20 h. Subsequently, the membranes were thoroughly rinsed with distilled water until a neutral pH was reached and then dried at 100 °C. The overall preparation process is illustrated in Figure 1. Uncross-linked membranes are denoted as PVA/MWCNTs, while cross-linking membranes are denoted as X-PVA/*n*MWCNTs, where *n* refers to the MWCNTs loading.

**Figure 1:**
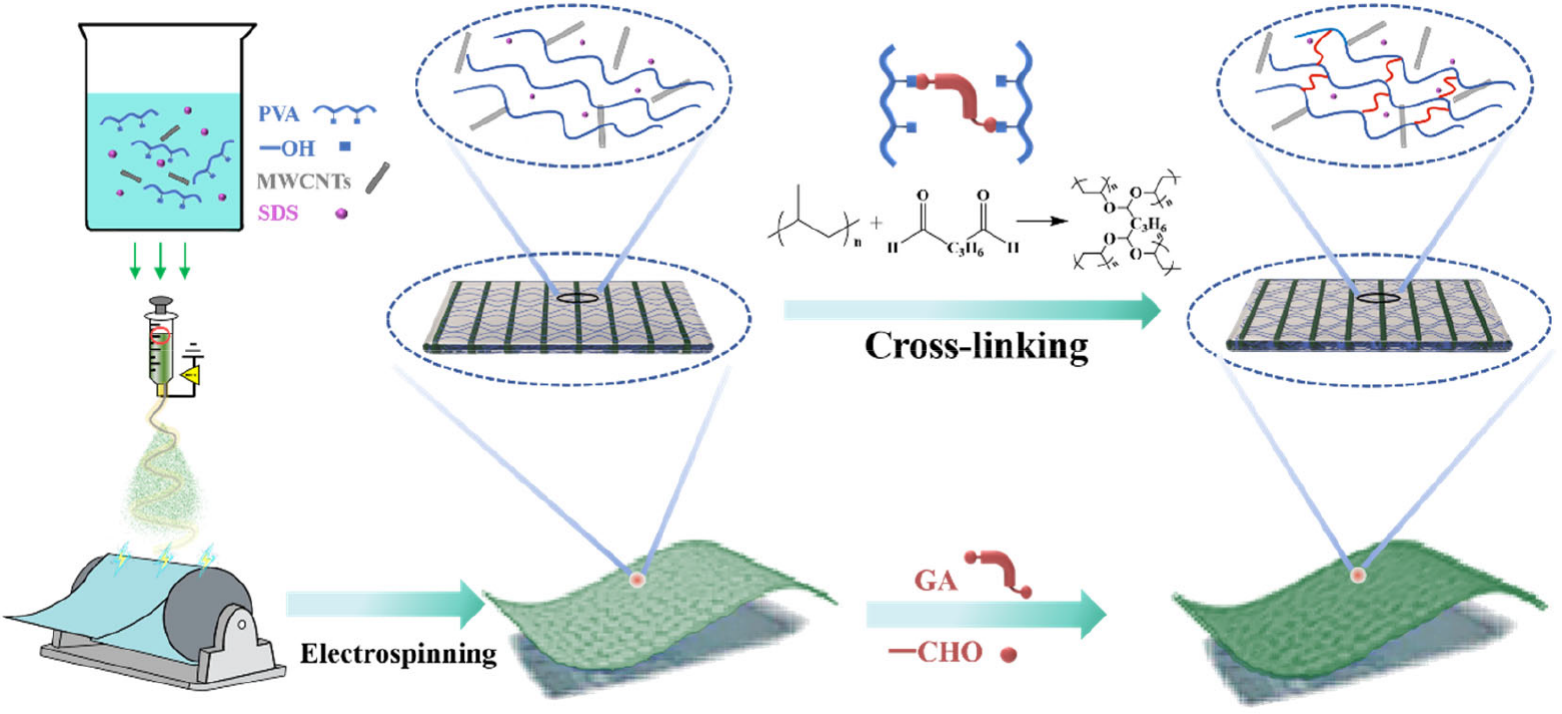
Preparation diagram of nanofiber membranes.

### 2.3 Characterization

The morphologies of the nanofiber membranes were examined using a ZEM20 scanning electron microscope (SEM) after gold sputter-coating. The internal dispersions of the membranes were observed by transmission electron microscopy (TEM, Tecnai F20). Fourier transform infrared spectroscopy (FTIR, 8400S) was conducted in a range of 4000–500 cm^–1^ with a spectral resolution of 4 cm^–1^. X-ray diffraction (XRD) patterns were recorded on an ARL X’TRA diffractometer using Cu Kα radiation (λ = 1.5409 Å) at a scanning rate of 5°/min over a 2θ range of 5–50°. Mechanical properties were measured with a CMT-424 electronic universal testing machine at a crosshead speed of 20 mm/min, and the reported values represent the average of at least five independent measurements. Surface wettability was characterized by static water contact angle measurements using a JY-PHb contact angle analyzer, and the average contact angle was determined from multiple measurements. The electrical resistance of the membranes was evaluated by using a CXT6015 insulation resistance tester under different environmental conditions. Specifically, samples were subjected to (i) washing cycles (10, 20, 30, 40, 50, and 100) in aqueous solutions with pH values of 1 (HCl-adjusted), 7 (deionized water), and 13 (NaOH-adjusted); (ii) ultraviolet (UV) irradiation (60 W) for 20–100 h in 20 h intervals; (iii) temperature variation from 0 to 95 °C in increments of 5 °C; and (iv) relative humidity variation from 10% to 90% in 10% intervals.

## 3 Results and Discussion

### 3.1 Micromorphology of Membranes

The SEM images and corresponding fiber diameter distributions of the nanofiber membranes are listed in Figure 2. The PVA/0.8MWCNTs membrane exhibited a nonuniform fiber diameter distribution with discontinuous structures (Figure 2a). With an increasing MWCNTs content, the fibers displayed smoother surfaces and larger average diameters (Figure 2b,c). This phenomenon can be attributed to the enhanced conductivity of the spinning solution provided by MWCNTs, which stabilized the Taylor cone and facilitated more uniform jet stretching under the applied electric field.^26^ In contrast, the cross-linking nanofiber membranes (X-PVA/*n*MWCNTs) exhibited a smoother surface, higher fiber-packing density, and larger average fiber diameter (Figure 2d–f). These results suggest that the cross-linking reaction further repaired the surface morphology and promoted the formation of more compact fiber networks.^27^

**Figure 2:**
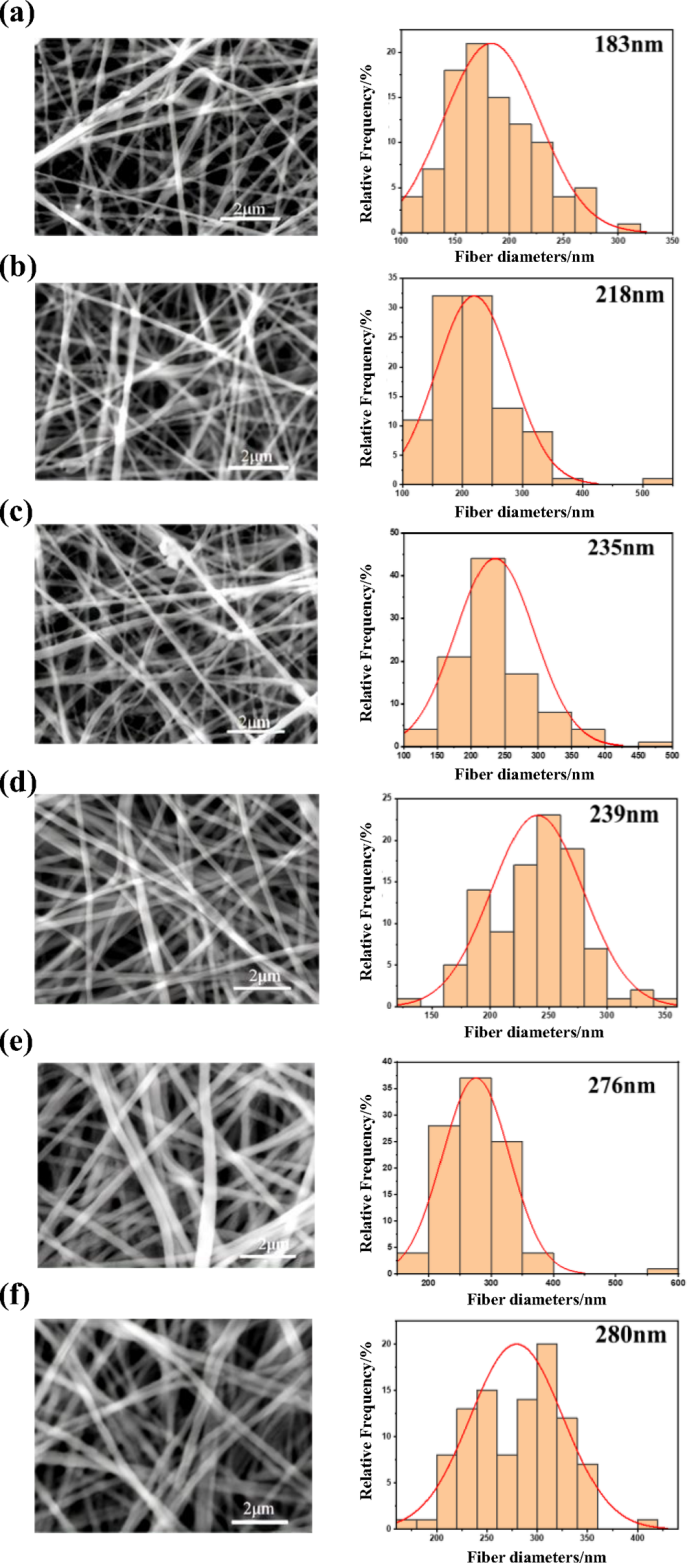
SEM and fiber diameter distribution images of nanofiber membranes. (a) PVA/0.8MWCNTs, (b) PVA/1.2MWCNTs, (c) PVA/1.6MWCNTs, (d) X-PVA/0.8MWCNTs, (e) X-PVA/1.2MWCNTs, and (f) X-PVA/1.6MWCNTs.

The internal dispersion of MWCNTs within the fibers was further examined by TEM (Figure 3). The nanotubes were embedded within the PVA matrix and predominantly aligned along the fiber axis (Figure 3a–c). Distinct lattice fringes observed in Figure 3d confirmed that the MWCNTs retained their polycrystalline structure during electrospinning, indicating that the process did not disrupt their intrinsic crystalline integrity.^28^ Furthermore, the localized aggregation of MWCNTs within the fiber matrix was observed in Figure 3e. This behavior could be attributed to the high specific surface area and surface energy of MWCNTs, which promoted aggregation and entanglement.^29^

**Figure 3:**
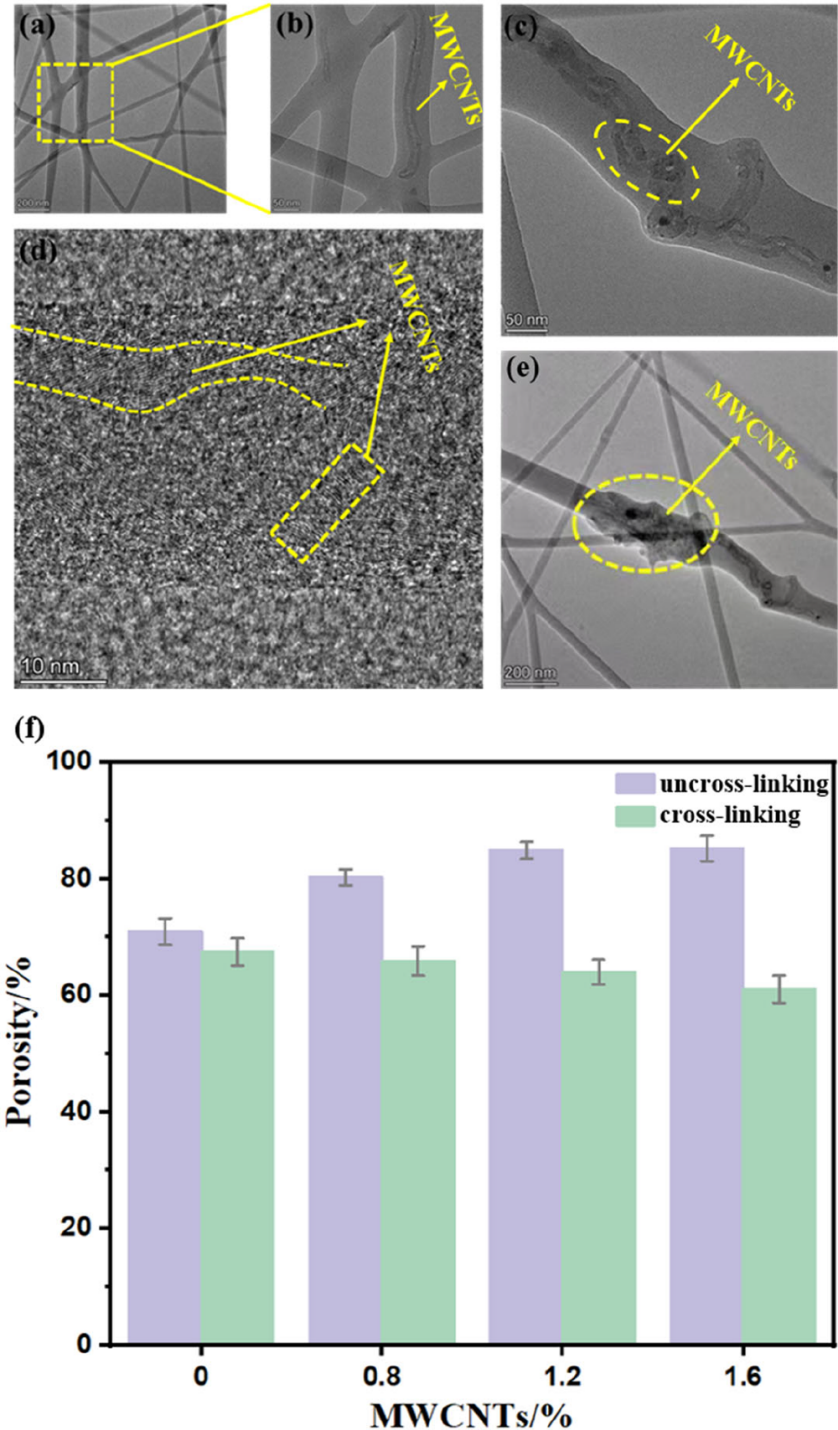
(a–e) TEM image of X-PVA/1.6MWCNTs at different multiples. (f) Nanofiber membrane porosity histogram.

As shown in Figure 3f, the porosity of PVA/*n*MWCNTs increased with increasing MWCNTs content, rising from 70.89% at 0.8 wt % to 85.21% at 1.6 wt %. In contrast, the porosity of X-PVA/*n*MWCNTs membranes decreased from 67.43% to 61.04% over the same filler range. This inverse trend can be attributed to cross-linking, which strengthened the interactions among PVA chains and led to the formation of denser fiber networks.

### 3.2 Structural Characterization

The FTIR spectra of the fiber membranes are presented in Figure 4a. The main absorption peaks remained essentially unchanged for both cross-linking and uncross-linked membranes with varying MWCNTs contents, suggesting that the incorporation of MWCNTs did not alter the characteristic chemical structure of the PVA matrix. Notably, in X-PVA/1.6MWCNTs, the O–H stretching vibration peak broadened and shifted from 3330 to 3444 cm^–1^ compared to PVA/1.6MWCNTs. This shift is attributed to the acetal reaction between the aldehyde groups of GA and the hydroxyl groups of PVA, which weakens hydrogen-bonding interactions among the PVA chains.^30^ In addition, the C–O stretching vibration peak observed at 1095 cm^–1^ in PVA/1.6MWCNTs was significantly attenuated or even disappeared in X-PVA/1.6MWCNTs, further confirming the consumption of −OH groups during cross-linking. Concurrently, two new absorption bands appeared at 1134 and 1008 cm^–1^, corresponding to the asymmetric and symmetric stretching vibrations of ether bonds (C–O–C) generated through GA-PVA cross-linking.^31^ These findings collectively confirm the successful formation of a cross-linking network structure.

**Figure 4:**
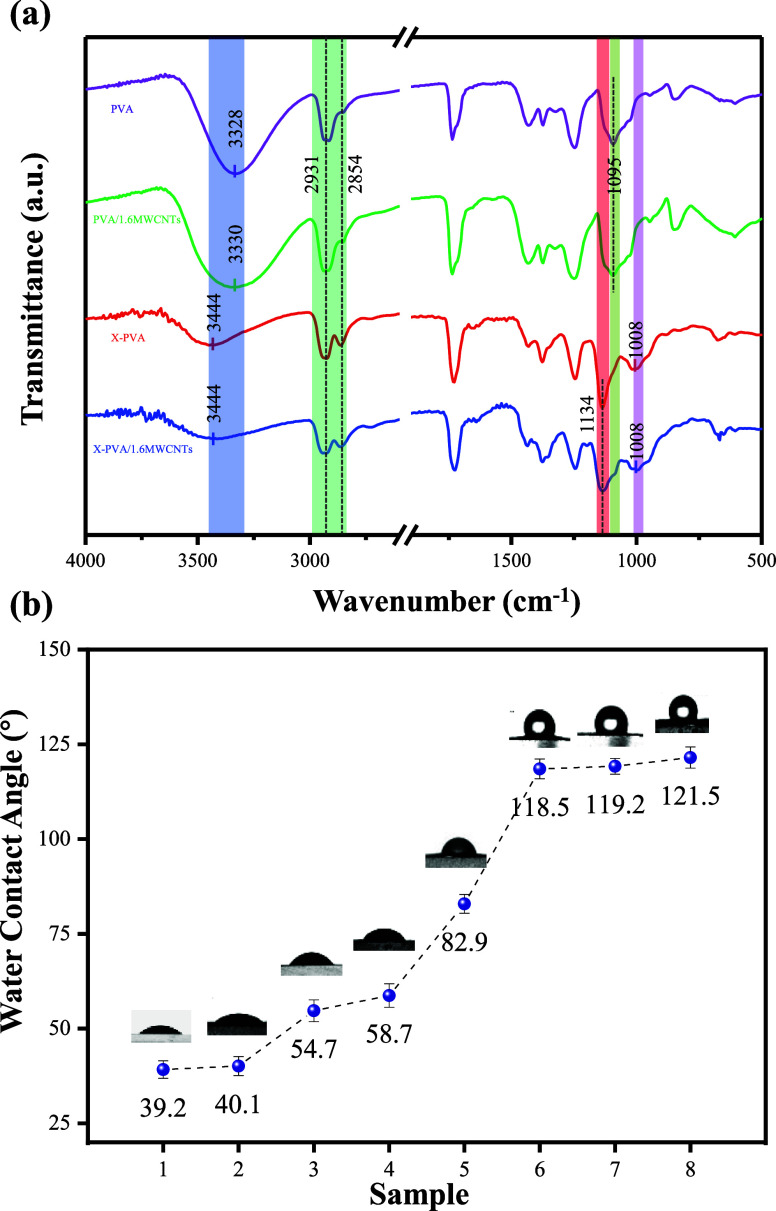
(a) FTIR spectra of nanofiber membranes. (b) Variation curve of water contact angle of nanofiber membrane (1: PVA, 2: PVA/0.8MWCNTs, 3: PVA/1.2MWCNTs, 4: PVA/1.6MWCNTs, 5: X-PVA, 6: X-PVA/0.8MWCNTs, 7: X-PVA/1.2MWCNTs, and 8: X-PVA/1.6MWCNTs).

The water contact angle measurements are listed in Figure 4b. Increasing the MWCNTs content progressively increased the contact angle of PVA/*n*MWCNTs membranes, indicating enhanced hydrophobicity resulting from the introduction of MWCNTs. Following GA cross-linking, the hydrophobicity was further improved, with the contact angle of X-PVA/1.6MWCNTs reaching 121.5°. This enhancement can be attributed to the consumption of hydrophilic −OH groups during cross-linking, which reduced surface polarity and thereby enhanced the overall hydrophobicity of the membranes. The XRD patterns (Figure S3) reveal that, compared with the pure PVA membrane, both the MWCNTs-containing and cross-linking samples exhibited decreased diffraction peak intensities, indicating a reduction in crystallinity. These results suggested that the incorporation of MWCNTs and the cross-linking treatment synergistically suppressed the crystallization behavior of PVA nanofibers.

### 3.3 Mechanical Performance

Figure 5 presents the stress–strain curves of the nanofiber membranes. The tensile strength of the PVA/*n*MWCNTs increased with higher MWCNTs loading. As shown in Figure 5 and Table 1, PVA/1.6MWCNTs exhibited a tensile strength of 11.0 MPa and a Young’s modulus of 132.1 MPa, corresponding to enhancements of 323% and 545%, respectively, compared with the pure PVA membrane. The remarkable improvement in tensile strength is attributed to the effective role of MWCNTs as a reinforcing phase, which enhances stress transfer and restricts the mobility of molecular chains within the PVA matrix. Moreover, the inherently high modulus of MWCNTs directly contributed to the substantial increase in Young’s modulus. After cross-linking, the Young’s modulus of the X-PVA/1.6MWCNTs further increased to 315 MPa, which can be ascribed to the restricted polymer chain mobility imposed by the cross-linking network, thereby enhancing overall rigidity. However, the formation of covalent cross-links also reduced chain mobility and plastic deformation capacity, resulting in a lower elongation at break in the cross-linking membranes.

**Figure 5:**
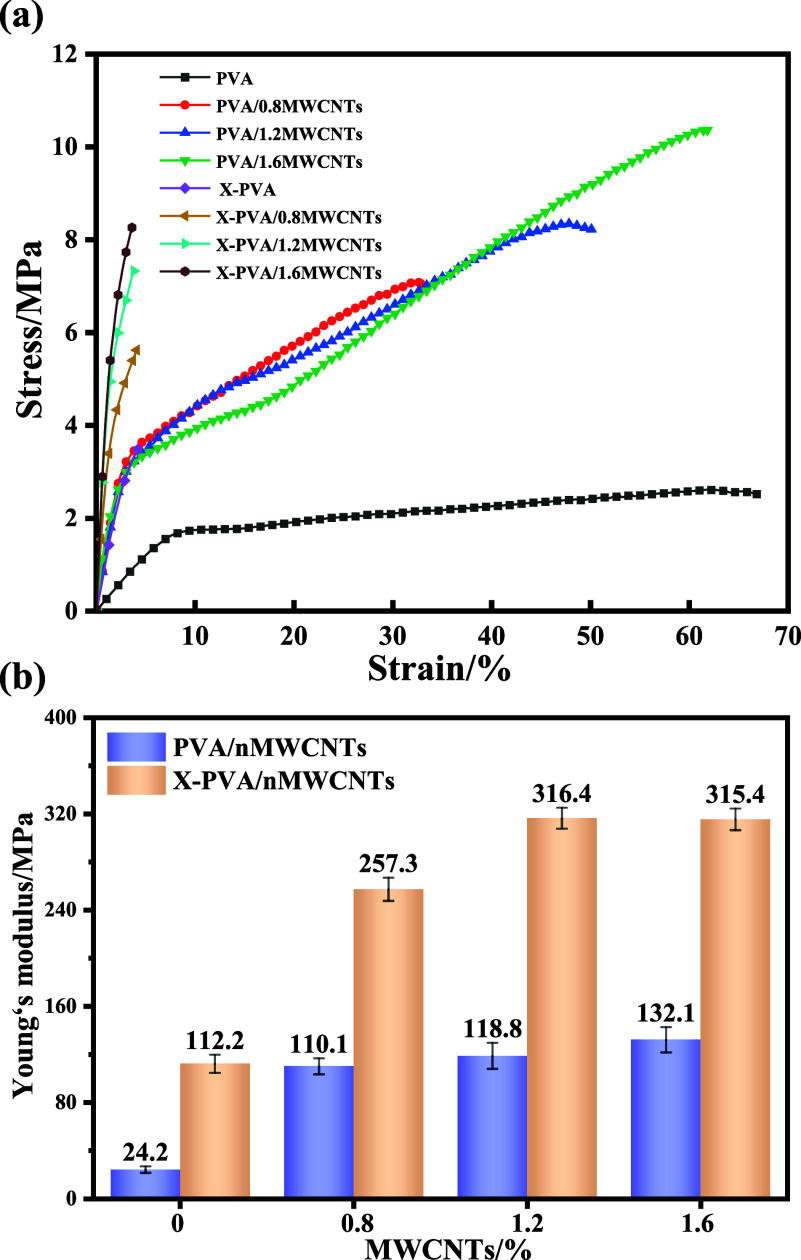
(a) Stress–strain curves and (b) Young’s modulus of the nanofibrous membranes.

**Table 1:** Physical and Mechanical Properties of the Nanofiber Membranes

sample	tensile strength (MPa)	elongation at break (%)	Young’s modulus (MPa)
PVA	2.6 ± 0.1	70.3 ± 5.5	24.2 ± 2.7
PVA/0.8MWCNTs	7.1 ± 0.2	37.0 ± 2.0	110.1 ± 6.6
PVA/1.2 MWCNTs	8.3 ± 0.3	54.4 ± 3.7	118.8 ± 10.8
PVA/1.6MWCNTs	11.0 ± 0.8	66.1 ± 4.3	132.1 ± 10.5
X- PVA	3.5 ± 0.1	4.2 ± 0.1	112.2 ± 7.5
X-PVA/0.8MWCNTs	5.6 ± 0.3	4.2 ± 0.2	257.3 ± 9.6
X-PVA/1.2 MWCNTs	7.3 ± 0.2	4.0 ± 0.1	316.4 ± 8.7
X-PVA/1.6MWCNTs	8.3 ± 0.4	3.7 ± 0.1	315.4 ± 9.0

### 3.4 Electrical Performance and Environmental Stability

The solvent resistance of X-PVA/MWCNTs is critical for their practical use in chemically aggressive environments. As shown in Figure 6a, after 24 h of immersion in water, the mass retention rates of the X-PVA/0.8MWCNTs, X-PVA/1.2MWCNTs, and X-PVA/1.6MWCNTs membranes were 85.25%, 90.33%, and 91.25%, respectively. The incorporation of MWCNTs effectively reduced water penetration into the PVA matrix, thereby enhancing the structural stability of the membranes in aqueous media. Figure 6b displays the macroscopic morphology of the nanofiber membranes after extended ultrasonication and exposure to media with different pH values. After 120 min of high-frequency ultrasonication and 1 week of immersion in acidic, neutral, and alkaline solutions, the membranes remained intact without visible degradation, confirming their excellent resistance to ultrasonic and chemical attack.

**Figure 6:**
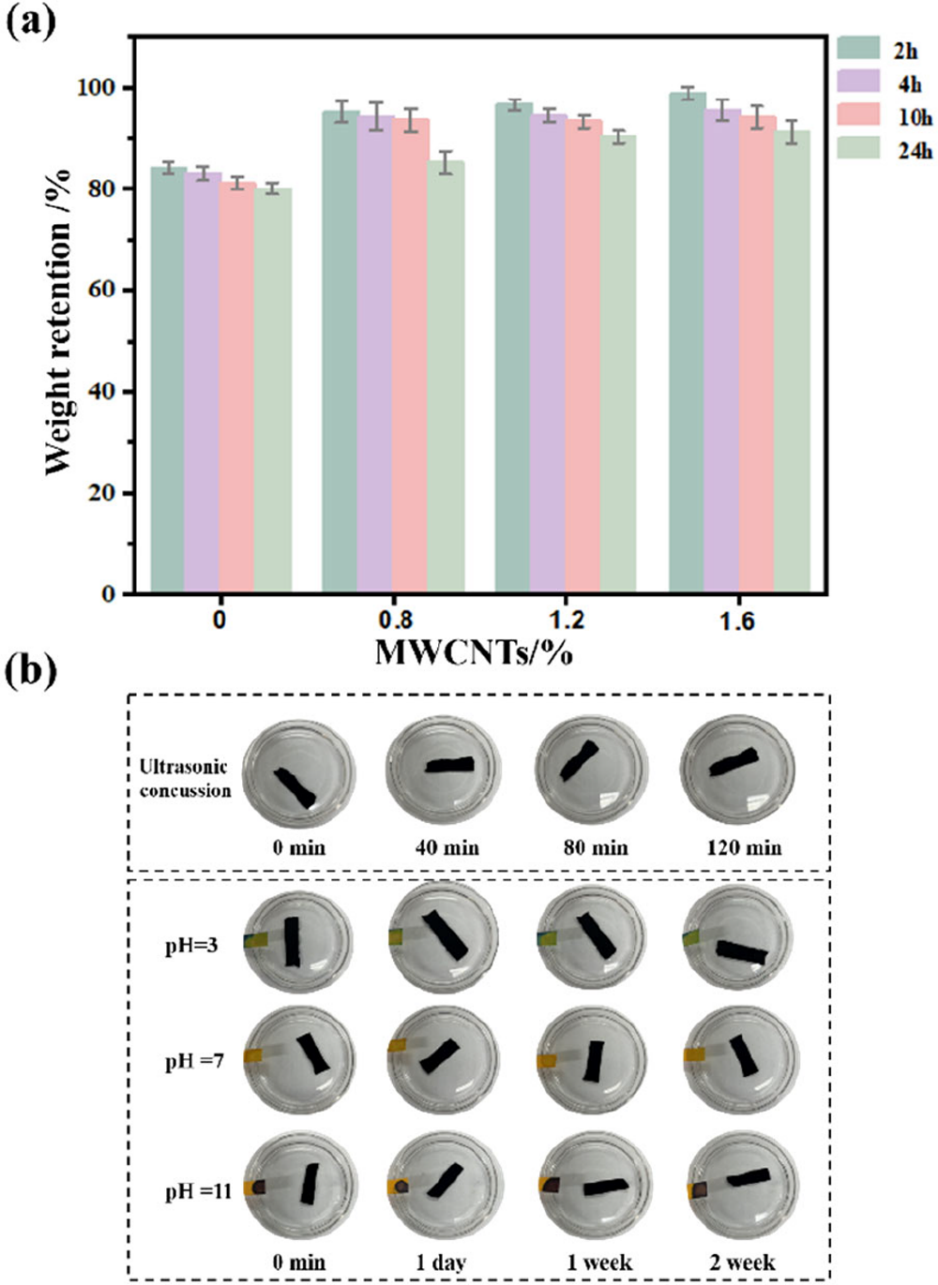
(a) Weight retention of X-PVA/*n*MWCNTs. (b) Structural stability of X-PVA/1.6MWCNTs at various pH levels.

The resistance of the nanofibrous membranes was investigated under various environmental conditions to evaluate the long-term environmental stability of their electrical performance. As shown in Figure 7a, the initial surface resistance of the cross-linking PVA membrane was 2.42 × 10^11^ Ω. Upon the incorporation of 0.8%, 1.2%, and 1.6% MWCNTs, the surface resistances of the corresponding X-PVA/*n*MWCNTs membranes decreased to 5.84 × 10^9^ Ω, 2.74 × 10^8^ Ω, and 6.65 × 10^7^ Ω, respectively, demonstrating that the addition of MWCNTs significantly improved electrical conductivity. The introduction of MWCNTs creates localized electronic states within the composite, which promote charge transport through hopping or tunneling mechanisms.^32^ Proper dispersion of MWCNTs is essential to preventing agglomeration and ensuring an effective percolation network.

**Figure 7:**
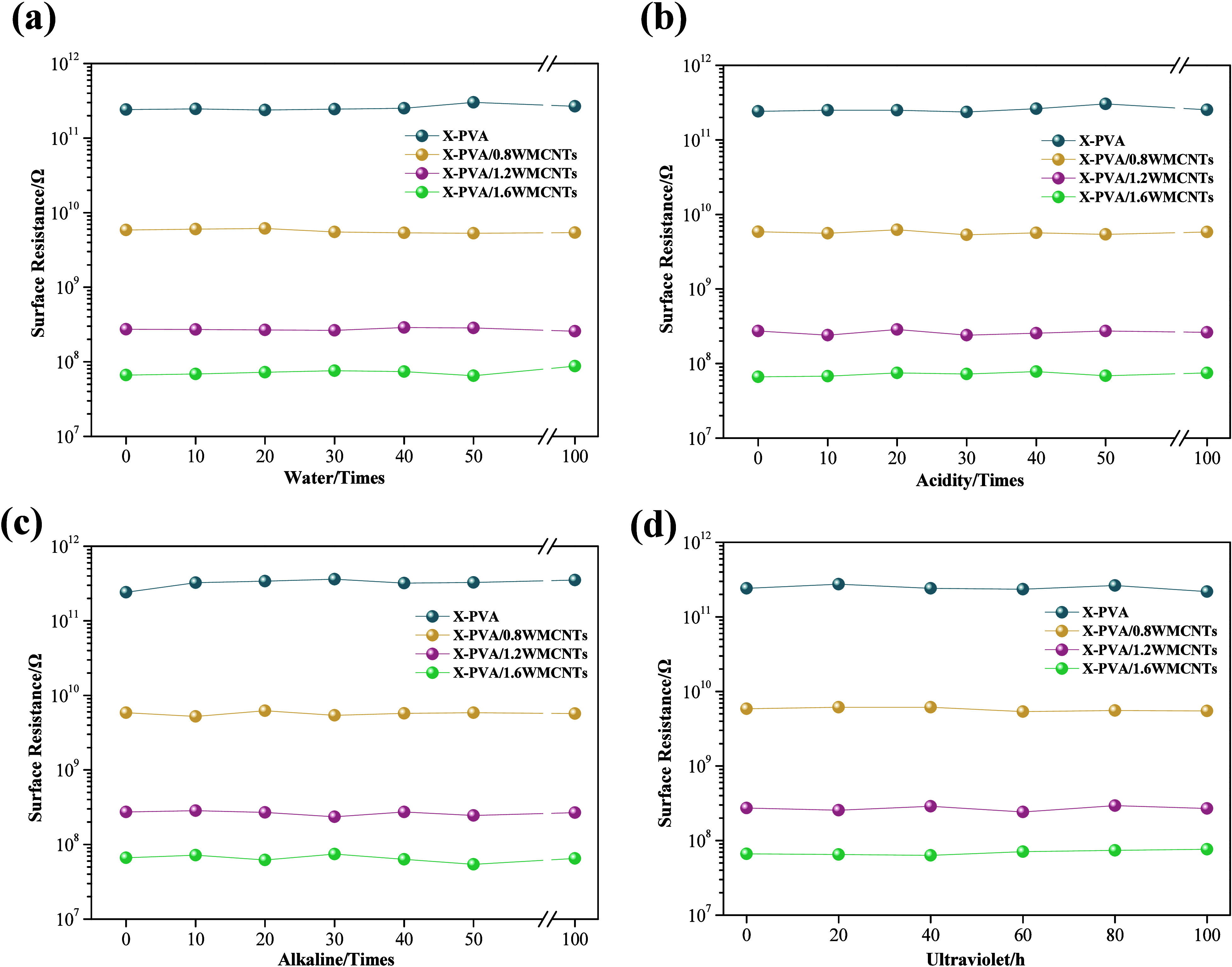
Electrical properties of cross-linking nanofiber membranes under different conditions. (a) Variation with washing cycles; (b) variation with acidic (pH 1) washing cycles; (c) variation with alkaline (pH 13) washing cycles; (d) variation with different ultraviolet radiation time.

The environmental stability of the fiber membranes was evaluated based on their electrical performance under different conditions. The X-PVA/MWCNTs maintained a stable electrical conductivity even after repeated washing cycles in strongly acidic (Figure 7b) and alkaline (Figure 7c) environments, demonstrating excellent acid and alkali resistance. Figure 7d and Figure S4 illustrate the variation in electrical resistance of the X-PVA/*n*MWCNTs under different UV exposure durations and temperature conditions, respectively. The results demonstrated that the membranes maintained a highly stable electrical performance upon exposure to both UV irradiation and thermal variations. As shown in Figure S5, the surface resistance of X-PVA/*n*MWCNTs decreased with increasing relative humidity. This phenomenon can be attributed to the absorption of water molecules by the nanofiber membranes, which facilitate the formation of conductive pathways, thereby significantly enhancing the charge transport efficiency.

## 4 Conclusions

In summary, high-strength and conductive PVA/MWCNTs nanofiber membranes were successfully fabricated via solution electrospinning. PVA/1.6MWCNTs exhibited a tensile strength of 11.0 MPa and a Young’s modulus of 132.1 MPa, representing enhancements of 323% and 545%, respectively, compared with pristine PVA membranes. MWCNTs served as an effective reinforcing phase by bearing and transferring stress within the nanofiber network. Moreover, MWCNTs’ intrinsic electrical conductivity endowed the membranes with favorable electrical properties. Further cross-linking treatment established a stable cross-linking network, enabling the X-PVA/1.6MWCNTs nanofiber membrane to maintain excellent structural stability and electrical conductivity (6.65 × 10^7^ Ω) under acidic/alkaline conditions, temperature variations, and UV irradiation. The resulting nanofiber membrane holds promising potential for applications in tissue engineering.

## Supplementary Material



## Notes

Additional characterization data (FTIR, XRD, and electrical properties) and detailed performance tests (determination of porosity, water contact angle, and electrical properties) (PDF)

## References

[1] Liu K., Yan S., Liu Y., Liu J., Li R., Zhao L., Liu B. (2024). Conductive and alignment-optimized porous fiber conduits with electrical
stimulation for peripheral nerve regeneration. Materials Today Bio.

[2] Sun R., Lang Y., Chang M., Zhao M., Li C., Liu S., Wang B. (2024). Leveraging Oriented Lateral Walls of Nerve Guidance
Conduit with Core–Shell MWCNTs Fibers for Peripheral Nerve
Regeneration. Adv. Healthcare Mater..

[3] Cai Y., Wang P., Li Y., Tang T. W., Zhang L., Shu H., Wong H., Li Y., Li J., Arias A. C., Zhang C., Jin G., Huang Q., Luo Z. (2025). Triple-Cue-Guided
Multichannel Hydrogel Conduit to Synergistically Enhance Peripheral
Nerve Repair. ACS Nano.

[4] Yan L., Liu S., Wang J., Ding X., Zhao Y., Gao N., Xia Z., Li M., Wei Q., Okoro O. V., Sun Y., Nie L., Shavandi A., Jiang G., Chen J., Fan L., Weng Y. (2024). Constructing Nerve Guidance Conduit using dECM-Doped Conductive Hydrogel
to Promote Peripheral Nerve Regeneration. Adv.
Funct. Mater..

[5] Liao Y., Loh C. H., Tian M., Wang R., Fane A. G. (2018). Progress
in electrospun polymeric nanofibrous membranes for water treatment:
Fabrication, modification and applications. Prog. Polym. Sci..

[6] He R. J., Teng C. X., Kumar S., Marques C., Min R. (2022). Polymer Optical
Fiber Liquid Level Sensor: A Review. IEEE Sens.
J..

[7] Kamarudin S. H., Mohd Basri M. S., Rayung M., Abu F., Ahmad S., Norizan M. N., Osman S., Sarifuddin N., Desa M. S. Z. M., Abdullah U. H., Mohamed Amin Tawakkal I. S., Abdullah L. C. (2022). A Review on Natural Fiber Reinforced Polymer Composites
(NFRPC) for Sustainable Industrial Applications. Polymers.

[8] Chen Y. J., Dong X. T., Shafiq M., Myles G., Radacsi N., Mo X. M. (2022). Recent Advancements
on Three-Dimensional Electrospun Nanofiber Scaffolds
for Tissue Engineering. Advanced Fiber Materials.

[9] Zhang P., Feng Z., Yuan W. S., Hu S. W., Yuan P. (2024). Effect of
PVA fiber on properties of geopolymer composites: A comprehensive
review. Journal of Materials Research and Technology-Jmr&T.

[10] Adelnia H., Ensandoost R., Shebbrin Moonshi S., Gavgani J. N., Vasafi E. I., Ta H. T. (2022). Freeze/thawed
polyvinyl alcohol hydrogels: Present, past and future. Eur. Polym. J..

[11] Yang R., Li X. M., Wang X. H., Jia L. M. (2023). Preparation of PVA/Ag
antibacterial hydrophobic slow-release composite films with core-shell
structure by one-step method. Mater. Lett..

[12] Wang X. G., Li Y. C., Meng D., Gu X. Y., Sun J., Hu Y., Bourbigot S., Zhang S. (2023). A Review on Flame-Retardant Polyvinyl
Alcohol: Additives and Technologies. Polym.
Rev. (Philadelphia, PA, U. S.).

[13] Karimzadeh Z., Mahmoudpour M., Rahimpour E., Jouyban A. (2022). Nanomaterial based
PVA nanocomposite hydrogels for biomedical sensing: Advances toward
designing the ideal flexible/wearable nanoprobes. Adv. Colloid Interface Sci..

[14] Thai N. L. B., Beaman H. T., Perlman M., Obeng E. E., Du C., Monroe M. B. B. (2024). Chitosan Poly­(vinyl alcohol) Methacrylate Hydrogels
for Tissue Engineering Scaffolds. ACS applied
bio materials.

[15] Bercea M. (2024). Recent Advances
in Poly­(vinyl alcohol)-Based Hydrogels. Polymers.

[16] Xue H., Jin J., Tan Z., Chen K., Lu G., Zeng Y., Hu X., Peng X., Jiang L., Wu J. (2024). Flexible, biodegradable
ultrasonic wireless electrotherapy device based on highly self-aligned
piezoelectric biofilms. Sci. Adv..

[17] Devi L. S., Paily R., Dasmahapatra A. K. (2025). Platinum
embedded conducting polyaniline/polyvinyl
alcohol hydrogel for enhanced glucose biomolecule detection. Polymer.

[18] Xia Y. J., Zhang Z., Li K. F., Zhao S., Chen G. B., Fei Z. F., Li X. H., Gan Z. C., Li X. W., Yang Z. C. (2024). Lightweight and high-strength SiC/MWCNTs
nanofibrous
aerogel derived from RGO/MWCNTs aerogel for microwave absorption. Chem. Eng. J..

[19] Woo J. S., Jin A. H., Yun H. D., Yu J., Bae J. H., Kim S. W., Seo S. Y., Lee G. C., Hong S., Kim K. S., Han S. (2025). Effects of ultrasonication
on electrical
and self-sensing properties for fiber-reinforced cementitious composites
containing MWCNTs. Journal of Materials Research
and Technology-Jmr&T.

[20] Ma L. Y., Nie Y., Liu Y. R., Huo F., Bai L., Li Q., Zhang S. J. (2021). Preparation of Core/Shell Electrically
Conductive Fibers
by Efficient Coating Carbon Nanotubes on Polyester. Advanced Fiber Materials.

[21] Moon M., Mim S. R., Billah M. M., Masud A. K. M. (2025). Synthesis and
characterization of surface modified MWCNTs reinforced PVA composite
films. Heliyon.

[22] Farag O. F., Abdel-Fattah E. (2023). Synthesis
and characterization PVA/plasma-functionalized
MWCNTs nanocomposites films. J. Polym. Res..

[23] Shi S., Si Y., Han Y., Wu T., Iqbal M. I., Fei B., Li R. K. Y., Hu J., Qu J. (2022). Recent Progress in
Protective Membranes Fabricated via Electrospinning: Advanced Materials,
Biomimetic Structures, and Functional Applications. Adv. Mater..

[24] Khan K. U., Masroor S., Rizvi G. (2023). Electrospinning and electrospun based
polyvinyl alcohol nanofibers utilized as filters and sensors in the
real world. J. Polym. Eng..

[25] Grasso G., Zane D., Foglia S., Dragone R. (2022). Application of Electrospun
Water-Soluble Synthetic Polymers for Multifunctional Air Filters and
Face Masks. Molecules.

[26] Zheng Q. L., Xi Y. W., Weng Y. X. (2024). Functional
electrospun nanofibers:
fabrication, properties, and applications in wound-healing process. Rsc Advances.

[27] Wang S., Wang N., Kai D., Li B., Wu J., YEO J. C. C., Xu X., Zhu J., Loh X. J., Hadjichristidis N., Li Z. (2023). In-situ forming dynamic
covalently
crosslinked nanofibers with one-pot closed-loop recyclability. Nat. Commun..

[28] Su X., Wang R., Li X., Araby S., Kuan H.-C., Naeem M., Ma J. (2022). A comparative
study of polymer nanocomposites
containing multi-walled carbon nanotubes and graphene nanoplatelets. Nano Materials Science.

[29] Mohd
Nurazzi N., Asyraf M. R. M., Khalina A., Abdullah N., Sabaruddin F. A., Kamarudin S. H., Ahmad S., Mahat A. M., Lee C. L., Aisyah H. A., Norrrahim M. N. F., Ilyas R. A., Harussani M. M., Ishak M. R., Sapuan S. M. (2021). Fabrication,
Functionalization, and Application of Carbon Nanotube-Reinforced Polymer
Composite: An Overview. Polymers-Basel.

[30] Chang Y., Wang Z., Liu F. (2025). Tunable Crosslinked
Polyvinyl Alcohol/Polyethylene
Glycol (cPVA/PEG) Nanofiber Membranes with Enhanced Mechanical and
Hydrophilic Balance. Molecules.

[31] Zou D., Zhou Y. Z., Yan W. T., Zhou Y., Gao C. J. (2022). Boric acid-loosened
polyvinyl alcohol/glutaraldehyde membrane with high flux and selectivity
for monovalent/divalent salt separation. J.
Membr. Sci..

[32] Zare Y., Rhee K. Y. (2020). Calculation of tunneling distance in carbon nanotubes
nanocomposites: effect of carbon nanotube properties, interphase and
networks. J. Mater. Sci..

