# Supplementary material for: Conductive Poly(vinyl alcohol)/Multiwalled Carbon Nanotubes Nanofiber Membranes with High Environmental Stability
Source: ACS Omega. 2026 Feb 5;11(6):10683–90. doi: 10.1021/acsomega.5c12610 (PMC12917829; doi:10.1021/acsomega.5c12610)
Supplement: Supplementary file 1 [file ao5c12610_si_001.pdf]

Supporting Information

Conductive PVA/MWCNTs Nanofiber Membranes with High Environmental Stability

Hui Xiao[1, 2†], Hongyu Lin[1†], Jingyi Wang[1,3], Chuanli Yu[2], Huaxin Wang[1], Liqun Chen[3], Hongbing Jia[2*]*

1. School of New Materials and Shoes & Clothing Engineering, Liming Vocational University, Quanzhou, 362000, China
2. Key Laboratory for Soft Chemistry and Functional Materials of Ministry of Education, Nanjing University of Science and Technology, Nanjing 210094, China
3. HTT Material Technology CO. Ltd., Quanzhou, 362005, China
*Corresponding. jingleewong@gmail.com (Jingyi Wang), polymernjust@gmail.com (Hongbing Jia)
[†]These authors contributed equally to this work

Characterization

Scanning electron microscopy (SEM)

The morphology of the composite nanofiber film visualized under a SEM (AIS2100, Korea Siren Technology Co., Korea). The fibrous samples were sputter-coated with a thin gold-palladium layer under vacuum. 99 nanofibers in each SEM image were randomly selected and the fiber diameter was measured by ImageJ software.

Transmission electron microscopy (TEM)

The nanofibers were directly electrospun on a double-sided copper grid. The internal structure of electrospun nanofibers was observed under a TEM (Tecnai F20, FEI USA Ltd.,USA) at an accelerating voltage of 200 KV.

X-ray diffraction (XRD)

Nanofiber membranes were cut into circular samples with a diameter of 5 mm and placed onto the sample stage of the X-ray diffractometer for testing under a XRD (Advanced D8, Bruker GmbH, Germany). XRD analysis was performed on an ARL X'TRA X-ray diffractometer using Cu Kα adiation (l ¼ 1.5409 A) at a scanning rate of 5 °min -1 from 5 to 50.

Fourier Transform Infrared Spectroscopy (FTIR)

The infrared spectra of electrospun nanofibers were recorded on a FTIR spectrometer (8400S, Shimadzu Co., Ltd., Japan) over a wavenumber range of 4000 to 500 cm^{-1} with a scanning resolution of 4 cm^{-1}

Mechanical strength

The mechanical properties were tested on a universal electronic testing machine (CMT-424, Sansi Test Equipment Co.,Ltd. Shenzhen China). The nano-fiber film was cut into sample strips of 1cm×5cm and the stretching speed was maintained at a speed of 20 mm/min.

Determination of porosity

Determination of porosity is calculated by measuring the dry state mass using an analytical balance to obtain the apparent density ρ_1. Based on the standard density of polyvinyl alcohol ρ_2 (ρ_2=1.31g·cm^{-3}) and ρ_1, the porosity of the fiber membranes can be calculated according to the equation ($\varepsilon = \left(1-\frac{\rho_1}{\rho_2}\right)\times 100\%$).

Water contact angle

The water contact angle test is performed at room temperature using a water contact angle tester (SL200B,Lenglen Information Technology Co.,Ltd. Shanghai China). The water contact angle was measured over a range of 0° to 180° with an accuracy of ±0.1°.

Electrical performance under varied pH levels

The electrical energy test was performed on high resistance meter （YH8200, Yuehua Electronic Technology Co.,Ltd. Dongguan China by changing conditions (Ⅰ. Water wash: 10, 20, 30, 40, 50, 100 under the conditions of pH = 7, pH = 1 (HCl), pH = 13 (NaOH), respectively. Ⅱ. Light time: under the condition of UV light (60W), the irradiation time is 20, 40, 60, 80 and 100h respectively. Ⅲ. Temperature: Adjust the

temperature 0-95℃, and measure every 5℃ interval. Ⅳ. Relative humidity: adjust the relative humidity 10-90%, and measure every 10% relative humidity interval.)

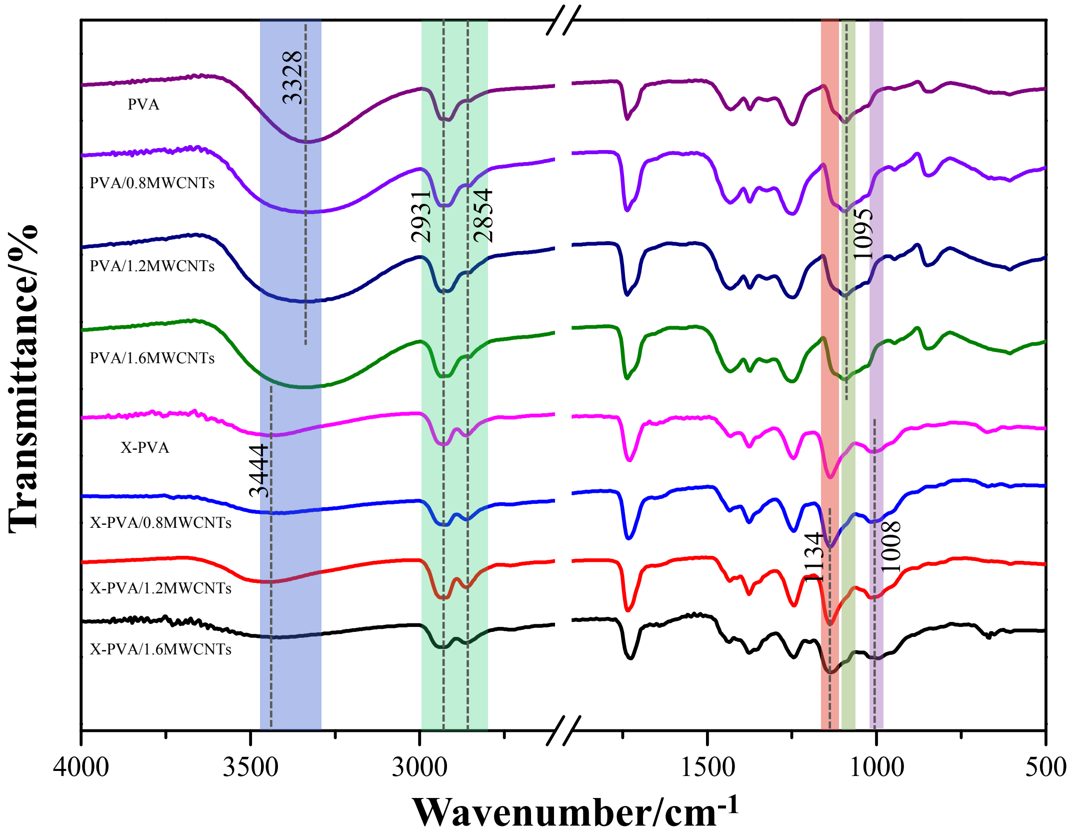

Figure S1. FTIR spectra of nanofiber membranes.

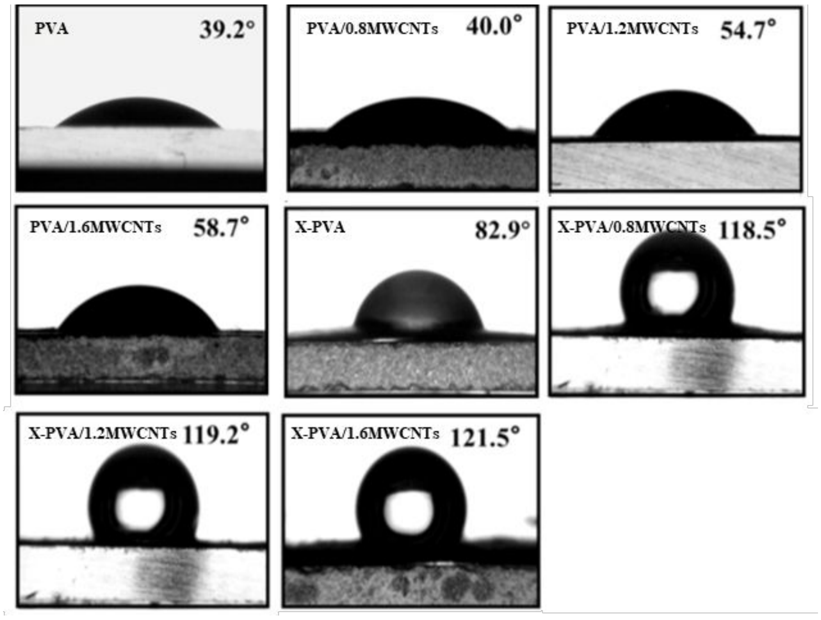

Figure S2. Water contact angle diagram of nanofiber membranes.

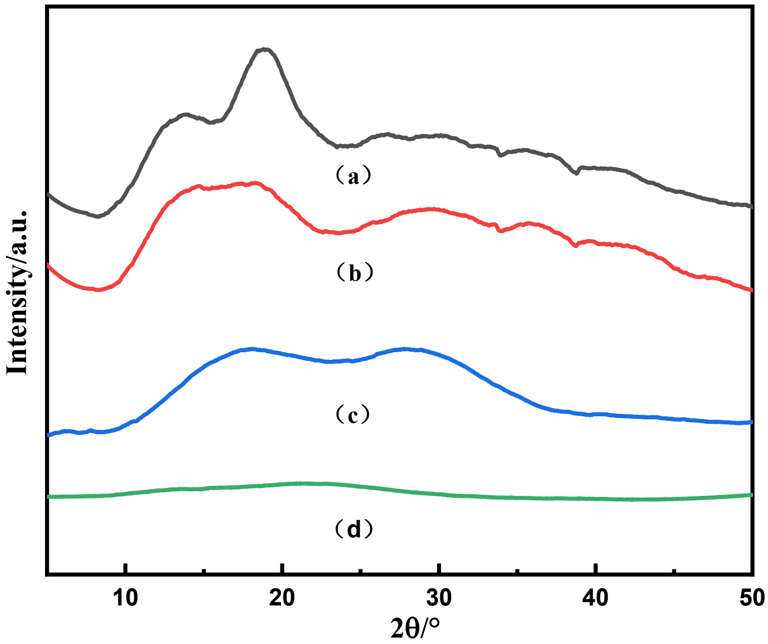

Figure S3. XRD diagram of nanofiber membranes. a:PVA, b:X-PVA, c:PVA/1.6MWCNTs, d:X-PVA/1.6MWCNTs.

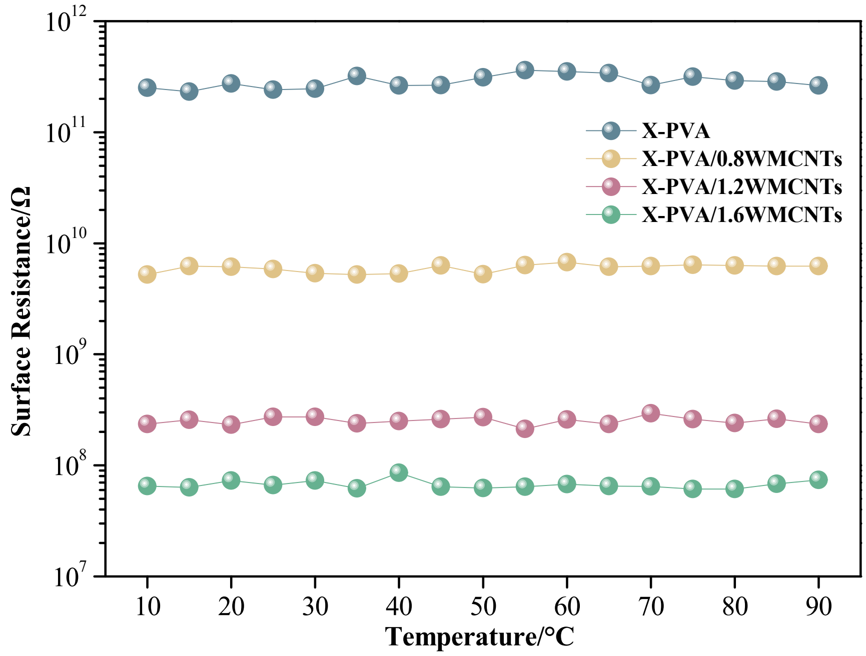

Figure S4. Electrical properties of crosslinked nanofiber films treated with UV at different times.

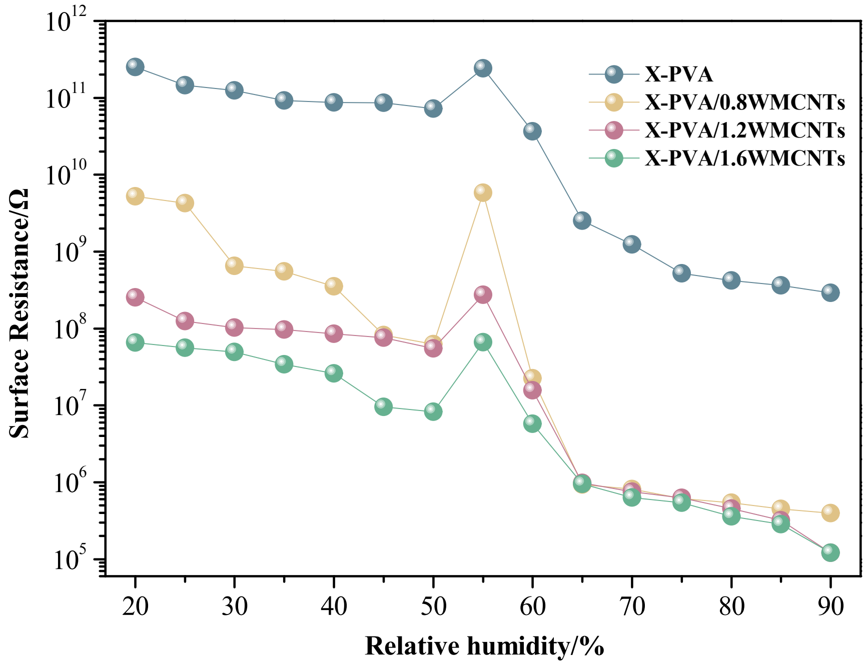

Figure S5. Electrical properties of crosslinked nanofiber films under different relative humidity.
